# Alexithymia and Internet Addiction: Mediating Role of Social Connectedness, Impulsivity, and Moderation by Depression

**DOI:** 10.5964/ejop.15353

**Published:** 2025-05-28

**Authors:** Utkarsh Utkarsh, Palak Kanwar

**Affiliations:** 1 Department of Psychology, Christ University, Bangalore, Karnataka, India; Lancaster University, Lancaster, United Kingdom

**Keywords:** internet addiction, alexithymia, impulsivity, social connectedness, depression

## Abstract

Internet addiction is a mounting concern in current times. Recent studies indicate a link between alexithymia and Internet addiction, but the underlying mechanisms of this association require more investigation. The present study explores the relationship between alexithymia and Internet addiction, with the mediating effect of Impulsivity and social connectedness, and the moderating effect of depression. A convenience sample of 362 participants between the ages of 18 and 25 years participated in this study and completed the Young’s Internet Addiction Test, Toronto-Alexithymia Scale, The Social Connectedness Scale, Barratt Impulsiveness Scale 15, and The Centre for Epidemiological Studies Depression Scale Revised. The results indicate that the direct effect of alexithymia on Internet addiction is partially mediated through impulsivity and social connectedness. Further, the moderating effect of depression is found to be non-significant. The results revealed two possible pathways through which alexithymia influences Internet addiction. Future research and interventions on Internet addiction can use these findings to mitigate the adverse outcomes of Internet addiction.

The Internet has become integral to modern life. Active Internet use is positively associated with subjective well-being (Verduyn et al., 2017), but its excessive and inappropriate use has adverse psychological and social effects (Diomidous et al., 2016; Kumar & Mondal, 2018). Internet addiction affects approximately 7.02% of the global population (Pan et al., 2020), with higher rates among some groups, such as college students in India, where 19.9–40.7% are at risk for Internet addiction (Joseph et al., 2021). Growing up in the digital age, emerging adults are especially prone to Internet addiction due to unregulated access, boredom, and poor time management (Wang, 2018), harming their physical health, academics, and emotional well-being (Elhai et al., 2021). Given the high prevalence of Internet addiction among emerging adults, it is essential to investigate the factors that contribute to its development.

The Diagnostic and Statistical Manual of Mental Disorders (DSM) does not formally recognise Internet addiction but includes Internet gaming disorder in Section 3 of its latest edition (DSM-5; American Psychiatric Association, 2013). Although researchers have proposed various definitions for Internet addiction, there is no consensus on its diagnostic criteria, and its theoretical foundation remains unclear, making the inclusion of Internet addiction in diagnostic frameworks controversial and highlighting the need for further research (Király et al., 2015; Ryding & Kaye, 2018). Young and Rogers (1998) defined Internet addiction as prolonged Internet usage with difficulty in its regulation, leading to psychological, social, and academic challenges. For Bleakley et al. (2017), Internet addiction refers to compulsive Internet usage similar to clinically diagnosable behavioural and substance use addictions. Internet addiction is often associated with excessive preoccupation with the Internet, poor time management, irritability when interrupted online, and reduced real-world social interaction (Tikhonov & Bogoslovskii, 2015).

Various factors contribute to Internet addiction, including age, gender, impulsivity, loneliness, alexithymia, and neuroticism (Kuss et al., 2014; Luo et al., 2022). Additionally, it is often associated with depression, anxiety, and personality disorders (Ostovar et al., 2016; Zadra et al., 2016). Recent studies explore the role of alexithymia, a multidimensional personality trait, in the etiopathogenesis of addictive disorders (Luo et al., 2022). Alexithymia is characterised by impairment in cognitive-emotional processing, defined as an inability to identify and describe emotions, difficulty fantasising, and an externally oriented thinking pattern (Taylor et al., 1997). It is a stable personality trait in the general population, with around 10% of people having problematically high levels of alexithymia (Parker et al., 2008). Poor emotional insight, like in alexithymia, is associated with increased psychological distress and unhealthy coping mechanisms, such as emotion-focused and avoidance-based coping (Saikkonen et al., 2018; Yurtdaş Depboylu & Fındık, 2024). It may lead to negative physical and mental health outcomes, including poor sleep, eating issues, cognitive difficulties, depression, distress, and reduced sexual activity (Mattila et al., 2010).

The relationship between alexithymia and Internet addiction can be understood through the self-medication hypothesis, which states that addictive behaviours serve as maladaptive coping mechanisms to alleviate distressing emotions or experiences (Baker et al., 2004). Individuals with alexithymia, due to poor emotional awareness and regulation, are more vulnerable to developing compulsive and addictive behaviours (Matias et al., 2023). Several studies find a significant association between Internet addiction and alexithymia in various populations (Li et al., 2023; Luo et al., 2022). Comparative research also indicates that individuals with alexithymia score significantly higher on Internet addiction than those without alexithymia (Taylor et al., 2014), highlighting alexithymia as a key risk factor for Internet addiction (Mahapatra & Sharma, 2018). The compulsive and addictive Internet use in individuals with alexithymia is viewed as an attempt to self-regulate emotion regulation deficits (Gioia et al., 2021; Preece et al., 2023). It can contribute to emotional stability in individuals with alexithymia traits (Ko et al., 2012).

While the link between alexithymia and Internet addiction is established, its underlying mechanisms require further investigation. Mahapatra and Sharma (2018) reviewed multiple studies investigating the relationship between alexithymia and Internet addiction, along with potential underlying pathways. They examined factors such as childhood maltreatment, attachment disorders, temperament, personality, and self-concept and found that the causal direction of the relationship between alexithymia and Internet addiction remains unclear, as the impact of several other influencing variables has yet to be fully explored.

## The Mediating Role of Impulsivity

Impulsivity is defined as “a predisposition for a fast, unplanned reaction to internal or external stimuli without concern for the negative repercussions of these behaviours to oneself or others” (Moeller et al., 2001, p. 1784). Individuals with alexithymia tend to display more impulsive behaviours (Lyvers et al., 2014), which may be a key factor in the development of Internet addiction (Zhang et al., 2015). For instance, Cao et al. (2007) found that adolescents with Internet addiction exhibit increased impulsivity. For individuals with impulsivity, the Internet provides short-term rewards through gaming, surfing, or social networking, reinforcing immediate gratification (Diotaiuti et al., 2022). This impulsivity may be rooted in dysregulated emotional and behavioural responses that are characteristic of alexithymia (Herman et al., 2020; Schreiber et al., 2012).

Individuals with alexithymia often struggle with emotional regulation, which can lead to Impulsive behaviours, making them more vulnerable to the compulsive use of the Internet (Mahapatra & Sharma, 2018; Taylor et al., 1991). Additionally, Internet use is associated with sensation-seeking behaviour (Shaffer, 1996), suggesting that impulsive individuals may use the Internet as a tool for sensation-seeking. A recent study by Lyvers et al. (2021) aimed to study alexithymia, impulsivity, negative affect, and internet addiction symptoms in internet-using female university students and found that impulsivity mediates the relationship between alexithymia and Internet addiction. Therefore, a thorough exploration of the role of impulsivity in this relationship can reveal a pathway through which alexithymia increases the risk of Internet addiction.

## The Mediating Role of Social Connectedness

Social connectedness is an individual’s subjective awareness of having close relationships with the social surroundings (Lee & Robbins, 1995), which might be another factor in the relationship between alexithymia and Internet addiction. People with alexithymia have difficulty interacting and dealing with social environments and experience low social support (Karukivi et al., 2014; Vanheule et al., 2007). It can make them feel less connected to their social surroundings. Poor social connectedness is associated with compulsive Internet use, particularly when providing individuals with a more appealing alternative to offline social interactions (McIntyre et al., 2015). Compensatory Internet use theory suggests that individuals turn to the Internet to relieve stress from adverse life events (Kardefelt-Winther, 2014). Individuals with alexithymia often experience psychological distress (Yurtdaş Depboylu & Fındık, 2024), making the Internet a potential outlet for them to relieve stress, regulate emotions, and fulfil social needs (Matias et al., 2023). Social connectedness, hence, can play a possible role in the relationship between alexithymia and Internet addiction.

## The Moderating Role of Depression

Alexithymia is linked with deficits in emotional regulation, which can increase susceptibility to depression (Foran & O’Leary, 2013). There is high comorbidity between the two, with depression significantly explaining variance in alexithymia, as alexithymia scores change proportionally with depression severity, making depression an important variable to consider while studying alexithymia (Honkalampi et al., 2000). Similarly, Internet addiction and depression have a bidirectional relationship (Ye et al., 2023), with numerous studies suggesting a significant association between Internet addiction and depression (Bahrainian et al., 2014; Kumar & Mondal, 2018). Depression severity can interact with study variables and influence the relationship between alexithymia and Internet addiction.

## The Present Study

The present study investigates the relationship between alexithymia and Internet addiction in a sample of emerging adults, exploring the mediating roles of impulsivity and social connectedness and the moderating role of depression. Previous literature suggests that age and gender are related to Internet addiction (Ainin et al., 2017; Ostovar et al., 2016) and can influence the model through varied interactions. Similarly, the variance of age and gender is also observed in the levels of alexithymia symptoms (Mattila et al., 2006). To account for their potential influence on alexithymia and Internet addiction, age and gender were included as covariates in the model, ensuring that the primary focus remains on the effects of impulsivity, social connectedness, and depression.

**Figure 1:**
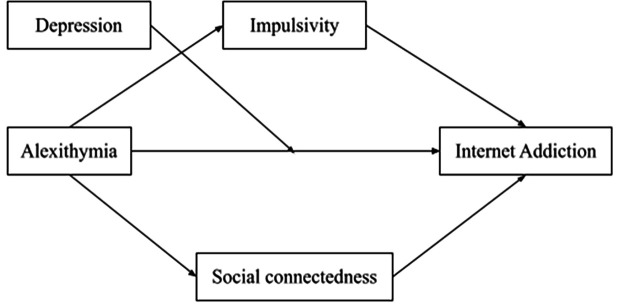
The Hypothesised Model

Accordingly, it is hypothesised that Impulsivity and social connectedness would mediate the relationship between alexithymia and Internet addiction after controlling for age and gender. The model is illustrated in Figure 1. Depression would moderate this relationship such that higher depression would strengthen the relationship between alexithymia and Internet addiction after controlling for age and gender.

## Method

### Design

This study adopts a quantitative method of inquiry and follows a cross-sectional design to examine the relationships between alexithymia, impulsivity, social connectedness, depression, and Internet addiction.

### Participants and Procedure

A total of 362 college students from India participated in this study, aged 18–25 years (*M* = 19.36; *SD* = 1.773). The sample consisted of 72.37% women (*n* = 262), 26.51% men (*n* = 96), and 1.10% non-binary individuals (*n* = 4). As part of the inclusion criteria, these students were required to be between 18 and 25 years old and residents of India, while individuals with psychological disorders were excluded from the study. Convenience sampling was used to recruit the participants, and the data was collected using an online survey with measures of alexithymia, Internet addiction, impulsivity, depression, and social connectedness after obtaining their informed consent. Participants also provided their socio-demographic details, and care was taken to maintain their anonymity to ensure confidentiality. The study followed ethical guidelines, ensuring compliance with the World Medical Association’s Declaration of Helsinki, and received approval from the institutional review board at CHRIST (Deemed to be) University, Bangalore. A total response rate of 95% was obtained.

### Measures

#### Internet Addiction

This study measured participants’ Internet addiction levels by Young’s Internet Addiction Test (IAT; Young, 1998). It is a 20-item scale that measures Internet usage in terms of the extent of preoccupation, inability to control Internet use, hiding or lying about Internet use, and continued use despite negative consequences of Internet use. Items include questions such as “How often do you find that you stay online longer than you intended?” and “How often do others in your life complain to you about the amount of time you spend online?” Items were rated on a 6-point Likert scale (0 = *Not applicable* to 5 = *Always*). The possible score ranges from 0–100, with higher scores indicating greater addiction. For the present sample, Cronbach’s alpha of the scale was found to be substantial (α = .904).

#### Alexithymia

Toronto-Alexithymia Scale (TAS-20; Bagby et al., 1994) was used to measure alexithymia. TAS-20 is a 20-item scale consisting of three subscales: seven items on difficulty describing feelings, five assessing difficulties describing feelings, and eight for externally oriented thinking. Items include questions such as “I am often confused about what emotion I am feeling” and “I am able to describe my feelings easily”. The items were rated on a 5-point Likert scale (1 = *Strongly disagree* to 5 = *Strongly agree*), with higher scores indicating higher levels of alexithymia. The scores range from 20–100. The internal consistency of the scale of the current sample was acceptable (α = .799).

#### Social Connectedness

The Social Connectedness Scale (SCS; Lee & Robbins, 1995) measured social connectedness. It assesses the perceived sense of belonging, connection with the external social world, and interpersonal closeness with others. The SCS is an 8-item scale with a 6-point rating scale (1 = *Strongly disagree* to 6 = *Strongly agree*). Items include questions such as “I feel disconnected from the world around me” and “I feel so distant from people”. Higher scores indicate a higher sense of connectedness. The scores range from 8–48. Cronbach’s alpha for internal consistency (α = .914) indicates good scale reliability in the current sample.

#### Impulsivity

Barratt Impulsiveness Scale 15 (BIS-15; Spinella, 2007) was used to measure the impulsiveness of the participants. It is a shorter version of an earlier, extensively used 30-item version (Patton et al., 1995). It comprises 15 items rated on a 4-point Likert scale (1 = *Rarely* to 4 = *Always*). BIS-15 maintains the earlier measure’s three-factor structure (five items each): non-planning, motor impulsivity, and attention impulsivity (Spinella, 2007). Items include questions such as “I act on the spur of the moment” and “I say things without thinking”. The scores range from 15–60. The scale has adequate internal reliability for the current sample with adequate Cronbach’s alpha (α = .807).

#### Depression

The Centre for Epidemiological Studies Depression Scale Revised (CESD-R 10; Andresen et al., 1994) was used to assess symptoms of depression. It is a 10-item self-reported scale with a 4-point Likert scale (0 = *Rarely* to 3 = *All the time*). Items include questions such as “I was bothered by things that usually don’t bother me” and “I had trouble keeping my mind on what I was doing”. The total score is calculated by adding the scores of the ten items. A higher score means more depression symptoms. The scores range from 0–30. For the present sample, Cronbach’s alpha was adequate (α = .801).

### Data Analysis

The data was analysed using IBM SPSS (Version 28) for descriptive statistics and reliability. Pearson correlation assessed relationships between the study variables. After the preliminary analysis, Hayes PROCESS Macro (Version 4.2; Hayes, 2013), the SPSS software application, was employed for moderation and mediation analysis using 95% confidence intervals from 5,000 bias-corrected bootstrapping resamples.

## Results

The intercorrelations were tested for the study variables, and significant correlations were observed. Table 1 presents an overview of the findings. Alexithymia showed a significant positive correlation with Internet addiction (*r* = .383, *p* < 0.01), impulsivity (*r* = .426, *p* < 0.01), and depression (*r* = .512, *p* < 0.01). However, social connectedness was found to have a significant negative correlation with alexithymia (*r* = -.476, *p* < 0.01) and Internet addiction (*r* = -.395, *p* < 0.01).

**Table 1:** Descriptive Statistics and Correlations for Study Variables

Variable	*M*	*SD*	1	2	3	4	5
1. Alexithymia	52.40	11.43	–	–	–	–	–
2. Internet Addiction	40.50	17.16	.383**	–	–	–	–
3. Impulsivity	33.02	7.47	.426**	.426**	–	–	–
4. Social Connectedness	30.79	10.02	-.476**	-.395**	-.253**	–	–
5. Depression	14.19	5.81	.512**	.358**	.318**	-.596**	–

*Note*. *N* = 362.

**p < .01 (two-tailed).

**Figure 2:**
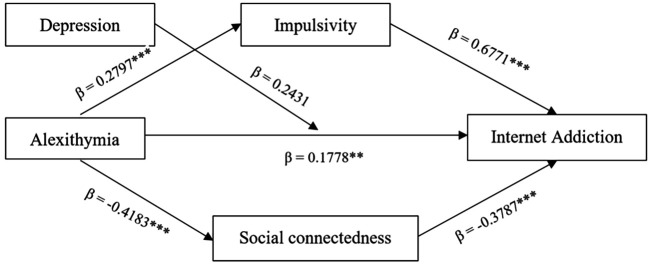
Standardised Estimates of the Model *Note. N* = 362. **p < .05. ***p < .001.

To test the mediating role of impulsivity and social connectedness and the moderating role of depression between alexithymia and Internet addiction, PROCESS Macro (Model 5) was used with age and gender as the controls in the model. The results indicated a significant direct effect of alexithymia on Internet addiction in the presence of impulsivity and social connectedness (β = 0.177, *p* < .05, 95% CL = 0.010/0.345). The effect of alexithymia on impulsivity was significant (β = 0.279, *p* < .05, 95% CL = 0.218/0.341), and impulsivity on Internet addiction was significant (β = 0.677, *p* < .05, 95% CL = 0.451/0.903), suggesting a significant indirect effect via impulsivity. Similarly, the effect of alexithymia on social connectedness was significant (β = 0.416, *p* < .05, 95% CL = -0.498/-0.337), and social connectedness on Internet addiction was significant (β = -0.378, *p* < .05, 95% CL = -0.575/-0.182), suggesting a significant indirect effect via social connectedness. Hence, both impulsivity and social connectedness partially mediated the relationship between alexithymia and Internet addiction. Depression did not significantly moderate the relationship (β = -0.010, *p* = .363, 95% CL = -0.032/0.012) (see Figure 2).

Moreover, the covariates age and gender had no significant effect on any predictor or outcome variable, indicating that they did not influence the model (see Table 2). Specifically, age did not significantly predict impulsivity (β = 0.129, *p* = .522, 95% CL = -0.267/0.529), social connectedness (β = 0.010, *p* = .968, 95% CL = -0.508/0.529), or Internet addiction (β = -0.613, *p* = .162, 95% CL = -0.248/1.475). Similarly, gender was not a significant predictor of impulsivity (β = -0.243, *p* = .753, 95% CL = -1.766/1.279), social connectedness (β = 0.642, *p* = .526, 95% CL = -1.347/2.631), or Internet addiction (β = -1.413, *p* = .405, 95% CL = -4.749/1.921).

**Table 2:** The Mediation and Moderation with 95% Confidence Interval

Predictor Variable	Outcome Variable	β	*SE*	*t*	*p*	95% CI [UL, LL]	
(Mediation and Moderation Effect)							
Alexithymia	Impulsivity	0.279	0.031	8.931	.000	0.218	0.341
Alexithymia	Social Connectedness	-0.418	0.040	-10.228	.000	-0.498	-0.337
Impulsivity	Internet Addiction	0.677	0.114	5.894	.000	0.451	0.903
Social Connectedness	Internet Addiction	-0.378	0.099	-3.795	.000	-0.575	-0.182
Alexithymia	Internet Addiction	0.177	0.085	2.086	.037	0.010	0.345
Depression	Internet Addiction	0.243	0.178	1.363	.173	-0.107	0.593
Depression*Alexithymia	Internet Addiction	-0.010	0.011	-0.909	.363	-0.032	0.012
(Control Variable Effect)							
Gender	Impulsivity	-0.243	0.774	-0.314	.753	-1.766	1.279
Age	Impulsivity	0.129	0.202	0.640	.522	-0.267	0.526
Gender	Social Connectedness	0.642	1.011	0.634	.526	-1.347	2.631
Age	Social Connectedness	0.010	0.263	0.039	.968	-0.508	0.529
Gender	Internet Addiction	-1.413	1.696	-0.833	.405	-4.749	1.921
Age	Internet Addiction	0.613	0.438	1.400	.162	-0.248	1.475

*Note. SE* = standard error; *CI* = confidence interval. *LL* = lower limit. *UL* = upper limit. 95% bootstrap CI with 5000 samples was used to estimate the direct, indirect, and interaction effects.

## Discussion

This study investigates the connection between alexithymia and Internet addiction, taking into account the roles of impulsivity, social connectedness, and depression. The findings indicate that alexithymia significantly influences Internet addiction, both directly and indirectly through impulsivity and social connectedness. However, depression does not moderate the association between alexithymia and Internet addiction.

First, the results showed a significant association between alexithymia and Internet addiction, supporting previous research with similar findings (Hao et al., 2019; Luo et al., 2022). A possible reason for this relationship could be that individuals with alexithymia exhibit deficits in emotional processing, contributing to psychological discomfort (Barbosa et al., 2011), impulsiveness (Herman et al., 2020), and low social connectedness, as evident in the present sample. Drawing from the Compensatory Internet use theory, these adverse emotional experiences and psychological distress faced by individuals with alexithymia may motivate them to turn to the Internet as a means of coping and relief (Kardefelt-Winther, 2014). The Internet offers instant gratification and emotional relief, making it a maladaptive coping mechanism for those with alexithymia, which can lead to addictive patterns of Internet use. Age and gender were included as control variables in the hypothesised model to account for their potential influence on the relationship between alexithymia and Internet addiction. However, neither age nor gender showed a significant effect, suggesting that the association between alexithymia and Internet addiction operates independently of these demographic factors. This finding aligns with previous research showing that alexithymia predicts Internet addiction independent of age and gender (Scimeca et al., 2014). However, other studies (Gori & Topino, 2023) suggest that demographic factors may influence this relationship, highlighting the need for further research.

Impulsivity was found to partially mediate the relationship between alexithymia and Internet addiction, which is consistent with the findings of Lyvers et al. (2021). Impulsivity’s mediating role can be explained as a pattern that stems from the emotional awareness and regulation difficulties in individuals with alexithymia, leading to ineffective emotional management strategies (Swart et al., 2009). Emotions guide behaviour by providing feedback and prompting reflection (Baumeister et al., 2007). However, individuals with alexithymia struggle to access their emotions, leading to dysregulated emotional and behavioural responses that can develop into impulsive traits (Herman et al., 2020). Notably, impulsivity strongly predicts Internet addiction (Tsai et al., 2020). The present study finds that impulsivity may be a factor explaining the relationship between alexithymia and Internet addiction. Addiction is a complex psychological and behavioural condition with many possible underlying processes (Simon & West, 2015). The Self-Medication Hypothesis (Khantzian, 1997) suggests that addictive behaviour serves as a response to self-regulation difficulties, where individuals attempt to manage and control negative emotions through self-medication. From this perspective, Internet addiction may be seen in individuals with alexithymia as a form of self-medication for dysregulated emotional and impulsive responses.

The findings further reveal that social connectedness partially mediates the relationship between alexithymia and Internet addiction. Individuals with alexithymia often struggle with social interactions (Vanheule et al., 2007) and experience a perceived lack of social support (Karukivi et al., 2014). This leads to feelings of disconnection from their social environment, causing interpersonal difficulties, including low-quality romantic relationships (Holder et al., 2015). Congruent with the Compensatory Internet use theory (Kardefelt-Winther, 2014), negative social experiences and low social connectedness may motivate individuals with alexithymia to engage in problematic Internet usage to fulfil unmet social needs and support (Zhang et al., 2018). Thus, the current findings validate the hypothesis that alexithymia can lead to Internet addiction via low social connectedness.

Contrary to the hypothesis, the study found that depression did not moderate the relationship between alexithymia and Internet addiction. While alexithymia is linked to a higher risk of depression (Bamonti et al., 2010; Sagar et al., 2021), not everyone with alexithymia and addiction shows signs of affective disorders (Morie et al., 2015). This suggests that there is variability in the role of depression between alexithymia and Internet addiction. Recent research supports that alexithymia predicts Internet gaming disorder and Internet addiction independently of depression (Pape et al., 2022). Hence, further research is needed to understand this relationship better.

### Limitations, Implications, and Future Directions

The present study provides insights into the alexithymia–Internet addiction relationship, but several limitations must be considered. Firstly, this study focused on a general measure of Internet addiction; however, future research should examine specific online activities such as social media use, online shopping, gaming, etc, to gain deeper insights into different patterns of addictive behaviour. Incorporating socio-economic and psychological factors into the model could provide a more comprehensive understanding of the underlying mechanisms driving Internet addiction. The cross-sectional design of this study prevents causal inferences, so future research should use longitudinal or experimental designs. The small, non-representative, and disproportionate sample, as well as the use of convenience sampling, limit the generalizability, so replicating the study with diverse populations is recommended. Internet-based, self-report questionnaires may introduce self-report bias; it is suggested that future research uses other forms of assessments to minimise this bias. Internet use is a heterogeneous behaviour encompassing a wide range of activities.

Nevertheless, this study makes a significant contribution to the literature. The findings enhance our understanding of how alexithymia relates to Internet addiction, highlighting the complex interplay of emotional, behavioural, and social factors. These insights can inform tailored interventions that address emotional awareness, impulse regulation, and social connectedness. Overall, this study enriches the literature and guides future research on the psycho-social factors in Internet addiction.

### Conclusion

This study explored the relationship between alexithymia, Internet addiction, impulsivity, social connectedness, and depression. Alexithymia had a significant effect on Internet addiction. Impulsivity and social connectedness partially mediated the relationship between alexithymia and Internet addiction, while depression did not moderate this relationship, and the effect of age and gender was not significant. These findings suggest a potential pathway through which individuals with alexithymia may develop Internet addiction, offering valuable insights for researchers and clinicians into the mechanisms underlying this relationship.

## Supplementary Materials

For this article, the following Supplementary Materials are available:

- Data. (Utkarsh & Kanwar, 2025)
- Code. (Utkarsh & Kanwar, 2025)
- Codebook. (Utkarsh & Kanwar, 2025)
- Study materials. (Utkarsh & Kanwar, 2025)

## Data Availability

The datasets collected and analysed during the current study, including codebook and materials, are available in the Open Science Framework repository (Utkarsh & Kanwar, 2025).
